# Patient-reported outcome and experience domains for diagnostic excellence: a scoping review to inform future measure development

**DOI:** 10.1007/s11136-024-03709-w

**Published:** 2024-06-08

**Authors:** Vadim Dukhanin, Mary Jo Gamper, Kelly T. Gleason, Kathryn M. McDonald

**Affiliations:** 1 Department of Health Policy and Management, Johns Hopkins Bloomberg School of Public Health, 624 N Broadway, Suite 643, Baltimore, MD 21205 USA; 2 Johns Hopkins University School of Nursing, Baltimore, MD USA; 3 Division of General Internal Medicine, Department of Medicine, Johns Hopkins University School of Medicine, Baltimore, MD USA

**Keywords:** Diagnostic excellence, Patient reporting, Health-related outcomes, Patients’ well-being, Measurement

## Abstract

**Purpose:**

“Diagnostic excellence,” as a relatively new construct centered on the diagnostic process and its health-related outcomes, can be refined by patient reporting and its measurement. We aimed to explore the scope of patient-reported outcome (PRO) and patient-reported experience (PRE) domains that are diagnostically relevant, regardless of the future diagnosed condition, and to review the state of measurement of these patient-reported domains.

**Methods:**

We conducted an exploratory analysis to identify these domains by employing a scoping review supplemented with internal expert consultations, 24-member international expert convening, additional environmental scans, and the validation of the domains’ diagnostic relevance via mapping these onto patient diagnostic journeys. We created a narrative bibliography of the domains illustrating them with existing measurement examples.

**Results:**

We identified 41 diagnostically relevant PRO and PRE domains. We classified 10 domains as PRO, 28 as PRE, and three as mixed PRO/PRE. Among these domains, 19 were captured in existing instruments, and 20 were captured only in qualitative studies. Two domains were conceptualized during this exploratory analysis with no examples identified of capturing these domains. For 27 domains, patients and care partners report on a specific encounter; for 14 domains, reporting relates to an entire diagnostic journey over time, which presents particular measurement opportunities and challenges.

**Conclusion:**

The multitude of PRO and PRE domains, if measured rigorously, would allow the diagnostic excellence construct to evolve further and in a manner that is patient-centered, prospectively focused, and concentrates on effectiveness and efficiency of diagnostic care on patients’ well-being.

**Supplementary Information:**

The online version contains supplementary material available at 10.1007/s11136-024-03709-w.

## Background

The diagnostic process is a complex, iterative, and collaborative activity with a goal to reduce diagnostic uncertainty and develop a more precise and complete understanding of a patient’s health problem [1]. Measuring and improving this complex process and its outcomes has been recognized as a priority only recently, so that diagnostic quality, in comparison, for example, with treatment, has been long overlooked [2]. Diagnostic excellence, an emerging construct, builds upon six aims of high-quality care—effectiveness, efficiency, timeliness, patient-centeredness, safety, and equity—as a departure point for this construct’s further exploration [3].

Traditional efforts to improve healthcare quality most often rely on measurements based on data supplied by clinical teams and administrative data, but patient reporting is increasingly collected as a valuable source of information [4]. Health-related patient-reported measures elicit either patient-reported outcomes (PROs) or patient-reported experiences (PREs). PROs are specific phenomena or underlying constructs of individual’s health status in defined populations, while PREs focus on patient care experiences encompassing whether something that should have occurred in a healthcare setting, has happened, or how often it has happened [5–7]. The scope of PROs and PREs relevant to diagnostic excellence is unknown, yet that knowledge could help refine, measure, and achieve such excellence [5]. Identifying diagnostically relevant PROs and PREs could foster research that increases the accuracy, validity, and timeliness of measurements of diagnostic care and provide researchers with tools to inform interventions that improve care and patients’ quality of life [6]. PROs and PREs have potential to both reflect and enrich the specification of the construct of diagnostic excellence as patient reporting brings the values, knowledge, context, actions, and power of patients and their care partners to the foreground [7, 8].

Given the need to understand patient-reported aspects in the emerging construct of diagnostic excellence, this study sought to conduct an exploratory analysis that would identify the needs for and inform the development of patient-reported measures for diagnostic excellence. Specifically, we aimed to: (1) describe the scope of diagnostically relevant PRO and PRE domains; and (2) review the state of measurement of these domains.

## Methods

Our exploratory analysis was an iterative and multi-pronged exercise where the findings of a scoping review of the literature were subsequently enhanced with targeted reviews in response to expert input and application of a published set of patient journey snapshots to validate diagnostic relevance of the domains. The exploratory analysis also included interactive activities at three expert convening sessions (see Figure). We maintained a prospective focus on diagnostic excellence, where PRO and PRE assessments could guide the diagnostic process moving forward, as opposed to after a diagnosis is established. We also were focused on effectiveness and efficiency of diagnostic care on overall patients’ well-being and health-related quality of life; thus, any domain described as specific only to a symptom or condition was excluded.

**Fig. 1:**
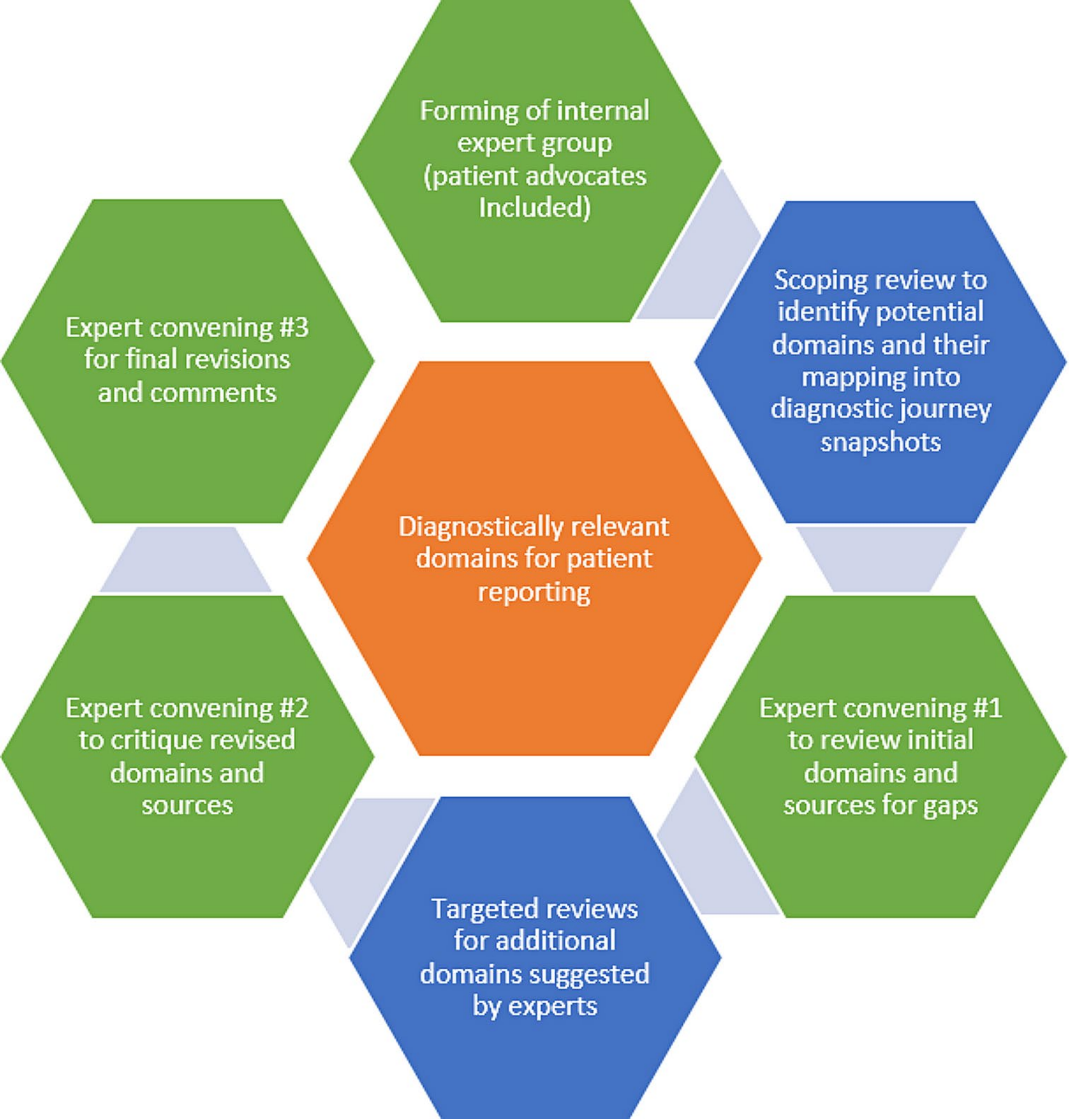
Elements of the study’s explanatory analysis

### Scoping review

The research team conducted a scoping review of a bibliographic database using multiple electronic search strategies designed to encompass patient outcomes, experiences, reflections, perceptions, or perspectives on any aspects of patient journeys that include or could relate to the diagnostic process and its outcomes. We followed the PRISMA-Scry (Preferred Reporting Items for Systematic reviews and Meta-Analyses extension for Scoping Reviews) checklist acknowledging the limitations of what this systematic approach to map evidence can offer and the need for expert inputs and iterative refinements [9, 10]. Due to a large amount of literature in this area (nearly 10,000 articles meeting inclusion criteria each year) and the level of resources available, we used only one database (EMBASE™ that covers all MEDLINE and additional articles [11]), select years of publication (past 1 to 5 years depending on a particular subtopic), and additional inclusion specifications (systematic reviews; explicitly diagnostic and screening contexts; caregiver and family focus; quality improvement or applicability contexts; descriptions of frameworks or stakeholder engagement). (See Supplemental Information for search terms used and search yields). The research team used similar searches for grey literature sources of national and international organizations concerned with patient-reported measures (see Supplemental Information). Subsequently, we used a backward snowballing approach (reviewing references in the earlier identified sources) and forward snowballing approach (reviewing publications that cited already identified sources) to find additional publications, including those prior to the year 2015 in cases where the earlier literature presented how the identified domain had been captured in a measure.

The research team shared and discussed literature findings with an internal expert group (8 researchers and 2 patient advocates in total) and at the expert convening (session #1). When additional PROs and PREs domains were emerging during these consultations, the research team conducted targeted searches to identify literature to supplement experts’ input. For example, the domain of accuracy of information was identified as initially missing, and the domain of feeling of being reassured was supplemented with examinations of false reassurance.

In the identified sources, the research team sought and extracted excerpts relevant to the contexts of diagnostic processes, procedures, and outcomes. Those individual excerpts formed a narrative bibliography. If the identified study did not already directly report measurement tools, we searched for other published examples of measures that assessed the identified domains. Our unit of analysis was an identified domain rather than a study. The research team did not intend to find all examples of measurement and was guided by saturation necessary for illustrative purposes to inform further deliberations.

### Diagnostic journey snapshots

To validate domains’ diagnostic relevance, the research team drew from the 2020 National Quality Forum report’s twelve snapshots of patient journeys containing diagnostic errors [12]. We then charted the identified domains onto these snapshots, which also facilitated clarification or identification of missing domains (see Supplemental Information for two examples).

### Expert convening

A total of 24 international experts participated in a series of five 2-hour virtual sessions in June 2021 (see Supplemental Information for expert bios). Participants were identified from author lists and other sources as having expertise related to either development of patient-reported measurement, analysis of diagnostic journeys, patient advocacy, patient safety interventions, communication, and equity and patient-centeredness of care. The experts further critiqued revised domains (session #2) and made final decisions on all identified domains (session #3); all sessions were facilitated by the research team that included a human-centered designer.

## Results

### Overview of diagnostically relevant PRO and PRE domains

The narrative bibliography amassed 41 diagnostically relevant and condition-agnostic PRO and PRE domains. While these domains were mostly described as patient-reported, we present these experiences and outcomes as those that can also be reported by care partners (e.g., family, friends, patient advocates, and others closely engaged in a patient’s care). Ten of these domains were classified as PRO, 28 as PRE, and 3 as mixed PRO/PRE. Based on identified examples of measurement practice, 27 domains are assessed immediately post-encounter with patients and care partners reflecting on a specific encounter(s) and 14 domains are assessed subsequently and cross-sectionally to reflect the entirety of the patient’s diagnostic journey and diagnostic care. Among these domains, 19 were captured in existing instruments (Table 1) and 20 in qualitative studies by eliciting free-text responses on questions corresponding to the domain concept (Table 2). Finally, one PRO domain and one PRE domain were conceptualized during this study, but the research team did not find examples of these domains being assessed (Table 3).

**Table 1:** Diagnostically relevant patient-reported outcome and experience domains captured by existing instruments

Domain Name	Experiences and/or Outcomes	Timing	Illustrative Example(s)
Respect (during the diagnostic encounter[s])	Experience	Immediately post-encounter(s)	During …, how often did [your doctor/nurse/allied health professional] treat you with courtesy and respect?[13]
Satisfaction (with diagnostic encounter[s])	Experience	Immediately post-encounter(s)	Using any number from 0 to 10, where 0 is the worst … care possible and 10 is the best … care possible, what number would you use to rate your care during this … visit?[14] Would you recommend this … to your friends and family? [Response options: Definitely no; Probably no; Probably yes; Definitely yes] [14]
Listening & taking concerns seriously	Experience	Immediately post-encounter(s)	During this … visit, how often did doctors/nurses listen carefully to you?[14] In a qualitative study [31]: If you had concerns related to …. Tell me about how your …. Providers discussed these with you: b. Did they ask questions? c. How did your providers address your questions?[31]
Duration of communication and Q&A	Experience	Immediately post-encounter(s)	How satisfied are you with the time your healthcare professional had for the conversation during which you were told ….? Give a number from 1 to 10 (1 = not satisfied at all, 10 = very satisfied)[16]. Did this [doctor/nurse/allied health professional] spend enough time with you?[13]
Emotional support at communication	Experience	Immediately post-encounter(s)	How satisfied are you with the emotional support from your healthcare professional during the conversation in which you were told …? (1 = not satisfied at all, 10 = very satisfied) [16].
Overall communication experience	Experience	Immediately post-encounter(s)	How would you rate the communication with your provider/the clinical team/hospital staff [17]?
Feeling of being reassured (including false reassurance)	Experience	Immediately post-encounter(s)	How do you feel now, at this moment, about the health concern that brought you to … [19]? [Response options: Not at all; Somewhat; Moderately so; Very much so] 3.1 I feel at ease 3.2 I feel concerned 3.3 I feel reassured 3.4 I have lingering concerns The Reassurance Questionnaire [18]: 3. If you initially feel reassured by a visit to your physician, does your anxiety return later on? 4. Do you keep worrying as long as it is not possible to rule out a serious illness? 5. Do you keep worrying as long as you do not know the origin of your symptoms? 8. Do you think your physician is keeping something from you? In a qualitative study [20]: “Repeated reassurance by primary health-care staff was the most common factor leading to a delay in referral. The parents of all the infants … were repeatedly reassured about the benign nature of the condition… Other health workers who gave false reassurance were …” [20]
Understanding of a health concern	Experience	Immediately post-encounter(s)	How you understand the health concern that brought you to …. Indicate how much you agree or disagree with the following statements. [Response options: Strongly disagree; Disagree; Neither Agree Nor Disagree; Agree; Strongly Agree] [19] 2.1 I have answers to all the questions I have related to my health concern. 2.2 I understand my health concern as much as I can at this point in time. 2.3. I have a clear picture or understanding of my health concern. 2.4 I have as much information as I need now.
Perception of a care plan for a patient to follow	Experience	Immediately post-encounter(s)	Thinking about the health concern that brought you to …do you… [Response options: Not at all; Somewhat; Moderately so; Very much so; There is nothing I need to do] [19] 4.1 Know what you need to do about the health concern (for example: what to watch for or treatment)? 4.2 feel you are able to manage the issue that brought you to the emergency department. 4.3. feel you have a plan you can follow. 4.4 feel you have figured out a plan. 4.5 know what to do if the issue got worse or came back. 4.6 know what you should be doing and/or not doing. 4.7 know what will happen next.
Emotional distress during diagnostic journey	Outcome	Immediately post-encounter(s)	Patient distress was assessed with the Distress Thermometer, a widely used measure of distress that is part of the National Comprehensive Cancer Network (NCCN) Distress Management Guidelines [21]. This brief screening tool rates overall distress (on a scale of 1–10), with scores > 4 suggestive of clinically significant distress. Change in distress was calculated as a difference score (-1 = distress increased, 0 = no change, 1 = distress decreased) [22]
Change in symptoms	Outcome	Immediately post-encounter(s)	How well do you feel you are recovering from the health concern that brought you ….? (Please check one) [19]. Completely better; Much improved; Slightly better; No change; Slightly worse; Much worse [19]. Patient-reported XXX {health concern} in the past week: None; intermittent; constant [23].
Perception of the symptom change trajectory	Outcome	Immediately post-encounter(s)	Which statement best describes you in the past 24 h (between this time yesterday and now)? I am better and have no more discomfort or symptom; I am better. I still have some discomfort or symptom, but I have figured out ways to avoid them; I am better. I still have some discomfort or symptom, but I can cope/live with them; I am not better at this point in time; I am worse [19].
Inaccurate diagnosis	Experience/Outcome	Subsequently and cross-sectionally	Thinking about the healthcare you have received … in the last 12 months, do you believe you had any problem related to … [Response options: No; Only once; More than once] [26] 1.1. Diagnosis of your problems? (e.g., wrong diagnosis) Thinking about the healthcare you have received … in the last 12 months, do you believe you had any problem related to… [Response options: No; Only once; More than once] [26] 1.7. Communication between you and the healthcare professionals ….? (e.g., not receiving the information you needed about your health problems or healthcare) In a qualitative study [27]: Inaccurate diagnosis: “In the past 5 years, has your provider given you the wrong explanation for your health care problem(s)?” Untimely diagnosis: “In the past 5 years, has it taken too long to receive an explanation for your health care problem(s)?” Failure to communicate diagnosis: “In the past 5 years, have you left … confused about the explanation of your health care problem(s)?”
Untimely diagnosis	Experience/ Outcome		
Failure to communicate diagnosis	Experience		
Types of harms	Outcomes	Subsequently and cross-sectionally	Do you think you have experienced any of the following types of harm as a result of the healthcare provided. in the last 12 months? [Response options: Not at all; Hardly any; Yes, somewhat; Yes, a lot; Yes, extreme] [26] 2.1. Pain. 2.2. Harm to your physical health. 2.3. Harm to your mental health. 2.4. Harm to your emotional health. 2.5. Increased limitations in doing your usual social activities. Do you think you have experienced any of the following types of harm as a result of the healthcare provided ….in the last 12 months? [Response options: Not at all; Hardly any; Yes, somewhat; Yes, a lot; Yes, extreme] [26] 1.1. Harm that led to increased healthcare needs (such as needed medications or tests). 1.2. Harm that led to increased personal needs (such as needing help preparing meals or cleaning). 1.3. Harm that led to increased financial needs. What harmful consequences have you experienced as a result of this error?[28] (a) mild allergic reaction; (b) severe allergic reaction; (c) other side effects; (d) deterioration of the health status; (e) unnecessarily prolonged pain; (f) wound infection / inflammation; (g) bleeding; (h) other part of the body has been injured; (i) a serious illness has not been recognized or has been recognized too late; (j) financial harm, e.g. additional therapy and treatment costs; k) temporal harm, e.g. you had to go to the practice again; l) mental or social harm; m) other harmful consequences. Please tell us, how severe or mild your (severest) harm was?[28] (a) very mild; (b) mild; (c) severe; (d) very severe. How long did it take to recover from (the severest) harm?[28] (a) less than a week; (b) more than a week but less than a month; (c) more than a month; (d) or has the harm remained permanent?[28] Due to this (severest) harm, did you …?[28] a) … go to see another physician? b) … call the medical on-call service / emergency service? c) … go to the emergency room? d) … go to the hospital overnight for treatment? e) … need rehabilitation?
Severity of harms			
Harms impact on recovery and/or permanence			
Disengagement with health systems / provider change	Outcome	Subsequently and cross-sectionally	Trust in Physician Scale (TPS) [30]: 1. I doubt that my doctor really cares about me as a person. 7. I feel my doctor does not do everything he/she should for my medical care. [Trust items were presented in a five-point Likert format, with response options ranging from “strongly agree” to “strongly disagree.“] Health Care Provider (HCP) Trust Scale [29]: My HCP: (6) Accepts me for who I am. (8) Treats me as an individual. I feel: (11) That other patients get better care from their HCPs. How often: (14) Do you think about changing to a new HCP? [Response Options: 0 = none of the time; 1 = some or a little of the time; 2 = occasionally or a moderate amount of the time; 3 = most of the time; 4 = all of the time].

**Table 2:** Diagnostically relevant patient-reported outcome and experience domains described qualitatively

Domain Name	Experiences and/or Outcomes	Timing	Illustrative Example(s)
Empathy & caring	Experience	Immediately post-encounter(s)	If you had concerns related to …. tell me about how your …. providers discussed these with you: a. Did they exhibit caring and empathy?[31]
Modality of communication, including telehealth	Experience	Immediately post-encounter(s)	“Information … was communicated using a variety of different modalities. Verbal information was communicated by health professionals, family, and friends and supplemented by audio-visual aids, angiogram images, anatomical heart models, information leaflets, Internet-based education, and graphical descriptions. Written information was considered to be less useful than other methods while participants were in-patients, mainly because they were often too exhausted to read, or the content was not sufficiently detailed or individualized.” [33]
Mode of communication materials and results			
Congruence with patient preference for autonomy in decision-making	Experience	Immediately post-encounter(s)	“This has created a tendency to oversimplify how prognosis [cancer diagnosis] is received (ie, only in relation to the patient vis-à-vis carers and/or family). Specifically, a singular emphasis on patients, and considering caregivers as virtual (and concurring) extensions of patients in terms of how prognosis is received. This is manifest in the (often implicit) logic of dealing with caregivers in conjunction with patients. While this protects patient autonomy, it also presupposes a degree of concordance between patients and their caregivers around what (and how much) they want to know about prognosis, as well as how they will react to this information.” [32]
Including family in communication			
Sufficient communication	Experience	Immediately post-encounter(s)	“the extent to which participants convey, extract, and exchange a sufficient amount of information in order to arrive at a shared understanding.” [46]
Clear communication	Experience	Immediately post-encounter(s)	“the extent to which care participants express and interpret verbal and nonverbal messages clearly (i.e. unambiguously) and utilize their interaction with each other to reduce uncertainty” [46].
Contextualized communication, including patient’s referral to additional information	Experience	Immediately post-encounter(s)	“the extent to which participants frame their interaction within local interactional circumstances such as hierarchies, time pressure, or discrepant goals that either facilitate or create barriers to shared understanding” [34]. If you had concerns related to …. tell me about how your …. providers discussed these with you [31]: c. Show you any materials?
Interpersonal adaptation, including cultural awareness and adaptation for health literacy level in communication	Experience	Immediately post-encounter(s)	If you had concerns related to …. tell me about how your …. providers discussed these with you: d. How did your providers address your concerns that might be different than other patients?[31] “the extent to which participants respond to implicitly (i.e. nonverbally) and explicitly (i.e. verbally) expressed needs and expectations to maximize the likelihood of shared understanding.” [34]
Safe, supportive, and comfortable environment for communication	Experience	Immediately post-encounter(s)	“The environment was also experienced as unpredictable and prone to quick change from boring to chaotic and intense… Another structure-related aspect was the experience of safety of the setting. Patients expressed a strong need to feel safe in the acute care setting, but primarily described unsafe environments that were experienced as prison-like…” [46]
Patient preference for who delivers diagnosis	Experience	Immediately post-encounter(s)	“When asked to rate their likelihood to use various communication methods to receive medical information, patients rated “talk to your doctor” highest, followed by “talk to your nurse”. In contrast, patients expressed relatively low likelihood to use digital communication, rating “text message” and “e-mail” lowest” [27].
Mitigating language barriers	Experience	Immediately post-encounter(s)	“The lack of an interpreter for patient participants who did not speak English as a first language was another important barrier” [33].
Providers’ checking of patient understanding	Experience	Immediately post-encounter(s)	“Patients also reported their dissatisfaction with doctors, indicating that the information given was not always adequate and that the doctors did not always check they had clearly understood the information.” [35]
Accuracy of information	Experience	Immediately post-encounter(s)	“the extent to which care participants convey correct information, interpret information correctly, and utilize their communication with each other to validate the accuracy of their communicated message content” [46]. “A problem or delay in gathering, understanding or interpreting the medical history, including: Wrong symptoms or main concern; Inaccurate diagnostic history; Inaccurate medical history; Wrong side; Wrong patient; Inaccurate social habits and circumstances; Inaccurate family history; Something important is missing; Inaccurate patient characteristics; Medical history, not further described.” [36]
Care partner/family involvement in diagnosis process	Experience	Immediately post-encounter(s)	“Caregivers’ accounts revealed instances where they felt shut-out of clinical appointments (by patients and/or health professionals), or where, while present in the consultation, they felt unable to participate, or not worthy of participation, in conversations. In these circumstances, caregivers recounted hesitations about their role and rights within the consultation, with a tendency to (reluctantly) sideline themselves within conversations about prognosis. While participants recognized the validity and importance of situating care and disclosure around patient need, they also described these times of exclusion and silences in and beyond therapeutic encounters as important sources of distress or apprehension.” [32]
Understanding of diagnostic process	Experience	Immediately post-encounter(s)	“While patients have expertise in their own experience of symptoms, they typically have minimal knowledge about their diagnostic journey to an unknown destination. The patient does not necessarily know what information is valuable for diagnosis, or when to be concerned that diagnosis is off track.” [7] “The active involvement of the patient can be a powerful mechanism to balance the cognitive course within the realm of the diagnostic process. However, this entails a change of paradigm, since the physician must be able to shift from the construction of an individual mental model to a shared mental model, according to the paradigm of distributed cognition. The patient and the doctor, in this way, would work as a small team, sharing information, purpose and decisions. The sharing and the co-construction of mental models to be used during the diagnostic journey is a mechanism for increasing the collective intelligence which acts as an error pre-emptive tool.” [37]
Experience of diagnostic uncertainty	Experience/Outcome	Immediately post-encounter(s)	If you had concerns related to … tell me about how your … providers discussed these with you [31]. f. Did talking about … cause you to worry or feel distress? g. How did your provider know if you were feeling these emotions? [qualitative analysis] This notion of appropriate emotional management of uncertainty… [31]
Disconnected diagnostic encounters across settings and providers	Experience	Subsequently and cross-sectionally	Overall, how would you rate your pathway until you were diagnosed with cancer?[38] Did you experience that there was a health professional with oversight and responsibility for your pathway … until you were given your diagnosis [38]? [Knowledge fragmentation across settings and time] In the last 6 months, how often did you have to repeat medical information that you had already provided during the same visit ?[39] [satisfaction with inpatient–outpatient coordination] How would you rate the overall coordination and teamwork between your regular outpatient doctor and the doctors who cared for you during your hospital stay ?[40]
Future attitudes and behaviors towards health systems and providers	Outcome	Subsequently and cross-sectionally	[qualitative analysis] Some patients recounted decreased confidence in the health care system and their clinicians after experiencing a diagnostic error: (1) “I lost faith in the surgeon because he didn’t tell me face to face.”; (2) “[I] go somewhere else for care.” Other comments noted, “I just don’t like doctors anymore.” [27]
Potential harms and their potential severity	Outcome	Subsequently and cross-sectionally	“… by asking patients whether an error occurred in … (yes, possibly yes, possibly no, or not at all). Perceptions of risk of harm from error were assessed using analog items, patients were requested to indicate the degree of health-related harm after these errors on a 7-point Likert scale ranging from extremely harmful (severe disability or death) to not harmful at all (no health consequences).” [41]

**Table 3:** Diagnostically relevant patient-reported outcome and experience domains that are conceptualized but not assessed

Domain Name	Experiences and/or Outcomes	Timing	Illustrative Example of Conceptualization
Awareness of pending diagnosis	Experience	Immediately post-encounter(s)	{conceptualized} Are you aware that the results of a diagnostic procedure … are pending?
Self-advocacy affirmation	Outcome	Subsequently and cross-sectionally	{conceptualized} As a result of …, how has your confidence level to advocate for own health changed? Did not change; I became less involved in my health {diminished self-advocacy}; I am more involved in my health; advocating for my own health became a very important part of my life {self-advocacy domineering over the rest of the patient well-being}.

### Domains captured in existing instruments

**Respect** is a well-established PRE domain, wherein patients and care partners assess their interactions with the care team during the diagnostic encounter. This domain is, thus, captured directly and immediately post-encounter, for example, by asking how often the patient felt treated with respect and courtesy by a specific provider or providers overall [13]. Questions assessing respect are part of existing instruments such as the Consumer Assessment of Healthcare Providers and Systems (CAHPS®) family of patient experience surveys [14].

**Satisfaction** is another well-established PRE domain that is tailored to reflect directly on a diagnostic encounter rather than the overall diagnostic journey. For instance, questions could ask patients or care partners to rate their visit on a scale from 0 (worst possible) to 10 (best possible) or the likelihood that they would recommend a provider to family and friends using a scale from “Definitely No” to “Definitely Yes” [15]. Our experts noted that collecting patient satisfaction during various stages of the diagnostic journey can mitigate concerns about ratings affected by the nature of received diagnoses (e.g., lower satisfaction when receiving “bad news”).

**Listening and taking concerns seriously**, **duration of communication**, and **emotional support at communication** are three PRE domains assessing specific qualities of provider communication during the diagnostic encounter. These three domains are captured by directly asking patients to assess frequency [15] or by Likert-type scales [16] evaluating these aspects of communication.

**Overall communication experience** is an umbrella PRE domain assessed immediately post-encounter; instead of inquiring into all specific communication-focused domains, an instrument might ask questions regarding communication as a whole [17]. Our experts noted that using such an approach to assessing communication is not desirable.

The **feeling of being reassured** is a PRE domain that encompasses both appropriate and false reassurance. Appropriate reassurance is assessed immediately post-encounter (e.g., via The Reassurance Questionnaire [18] or a question in Vaillancourt and colleagues’ instrument [19]). False reassurance is conceptualized in qualitative studies [20] but is not yet incorporated, for example, as an option describing patient feelings or suspicions among possible responses in reassurance assessments. It was noted that the timing for assessing a false reassurance versus timing to assess the presence of appropriate reassurance after the visit might preclude incorporating these two aspects in the same instruments.

**Understanding of a health concern** is a PRE domain captured immediately and directly post-encounter. As an example, questions ask the level of agreement with the statement “I have a clear picture or understanding of my health concern” [19]. The PRE domain **perception of a care plan for a patient to follow** is assessed similarly, for instance, as the level of agreement with a statement “I know what to do if the issue got worse or came back” [19]. However, it was noted that identified examples of this domain’s assessment do not examine patients’ attitudes towards the care plan or its alignment with their preferences, for example, preference for tradeoffs related to diagnostic procedures (e.g., invasiveness level vs. testing accuracy).

**Emotional distress** is a PRO domain that asks patients to rate their level of distress on their diagnostic journey, specifically attributing that distress to the diagnostic encounter. It may be captured via the Distress Thermometer [21] or other screening tools [22], including assessing the change in distress between several consecutive encounters. This domain is related to patients experiencing diagnostic uncertainty, described below, as the distress might be one of the outcomes of such uncertainty. However, distress might not be attributed exclusively to uncertainty and can be attributed to the diagnostic process as a whole.

The **change in symptoms** and related **perception of the symptom change trajectory** are two PRO domains captured immediately post-encounter [19]. Aligned with our experts’ suggestion, some assessments [23] specify that change in symptoms might not be just toward improvement or worsening but can have an intermittent pattern. Assessment of the perception of the symptom change trajectory is done, for instance, by asking patients to align themselves with statements that describe their understanding and accommodating to their health status trajectory in the past 24 h [19]. Alternatively, it might be inquired if the symptom change trajectory is perceived as expected or unexpected versus the diagnostic explanation received at the encounter. As noted by our experts, the assessment of change in symptoms may be potentially done via instrumental activities of daily living (IADLs) or other patient-reported measures of functioning [24].

The three domains **inaccurate diagnosis, untimely diagnosis,** and **failure to communicate diagnosis** are directly informed by the definition of the diagnostic error [25]. The first two domains capture both the outcome (diagnostic process resulted in a misdiagnosis or delay) and the experience (the patient has experienced delay or misdiagnosis). Failure to communicate diagnosis is classified as a PRE domain, given definitional debates about whether the communication gap stands alone as an error outcome. The literature reports on assessing these 3 domains subsequently by asking patients to reflect on the entirety of their diagnostic care (e.g., during the past 12 months). Two questions from the Patient Reported Experiences and Outcomes of Safety in Primary Care (PREOS-PC) [26] ask patients the number of instances they experienced problems related to their diagnosis and its communication. A qualitative study [27], directly elicited patient reports of inaccurate diagnosis, untimely diagnosis, and failure to communicate diagnosis via free-text responses.

Three PRO domains related to harms, specifically **type of harms, severity of harms, and harms impact on recovery and/or permanence,** were often assessed together (e.g., type of harm described via severity or severity of harm described via impact on patients) [26, 28]. These domains were captured cross-sectionally and asked patients about harms perceived as resulting from healthcare interactions, without attempts to attribute harms to particular providers, visits, or institutions. Our experts noted that harms should encompass not only harms due to diagnostic errors but harms resulting from diagnostic procedures, including overdiagnosis harms, which to date has not been incorporated into assessments.

**Patient disengagement** with the health systems, either as complete disengagement, not seeking care, or when patients are disengaged partly, for example, via changing healthcare providers, is a PRO that is not captured directly in the literature. Two instruments on trust in one’s healthcare provider [29] or physician [30] assess the domain indirectly, including the intention (frequency of ideation) to change providers. Exemplars of assessment are done in cross-sectional manner where a patient reports their attitudes toward a healthcare provider overall or even by reflecting on the entire care team involved.

### Domains described qualitatively

At the time of the analysis, we have not identified existing instruments capturing the remaining domains in diagnostic contexts. Table 2 provides excerpts of qualitative analyses or narrative descriptions of 20 such domains. Fourteen PRE domains reflect aspects of patient-provider communication assessed immediately post-encounter and are included in the overall communication experience domain described above. These domains are: **empathy and caring; modality of communication** (e.g., in person, written, or telehealth); **mode of communication** (e.g., presented visually or verbally); **congruence with patient preference for autonomy in decision-making; including family in communication; sufficient communication; clear communication; contextualized communication** (e.g., patient referred to additional information source); **interpersonal adaptation,** including cultural awareness and adaptation for health literacy level in communication; **safe, supportive, and comfortable environment** for communication; patient **preference for who delivers diagnosis; mitigating language barriers;** providers’ **checking of patient understanding;** and **accuracy of information.**

The overlaps between the content of some domain concepts are most evident in this group. For example, empathy and caring relate to the domains of respect and listening and taking concerns seriously [31]; congruence with patient preferences for autonomy in decision-making overlaps with inclusion of family in communication [32], but family roles have additional aspects beyond decision-making. Questions in existing instruments do assess PRE of providers’ ability to explain things in a way that patients could understand [15]. However, those questions do not differentiate clear communication from contextualized communication or from interpersonal adaptation in the diagnostic context and merge those domains when assessing. Other exemplars merge mode and modality of communication [33]. There are conceptual overlaps between, for example, sufficient communication (that implies sufficient understanding [34]) and understanding of a health concern or understanding of the diagnostic process. Similarly, the domain of clear communication [34] encompasses the resulting clarity of understanding of all medical information. Each of these examples and others can be argued as conceptually separate constructs in the diagnostic context, such as providers’ checking of patient understanding assesses whether this process occurred as part of the encounter [35] rather than the understanding itself. Accuracy of information has elements of patient-reported breakdowns [36], as patients report on accuracy of their medical records, as well as experiences being stigmatized and labeled by information that is inaccurate, outdated, or uses biased or judgmental language.

Aside from communication-focused domains, we identified six additional domains described qualitatively. **Care partner or family involvement** captures perceptions of how well healthcare systems facilitate care partner participation in diagnostic processes ranging from care partners feeling shut-out to equal partnership [32]. As noted by our experts, this domain includes family members and broadly defined care partners, including patient advocates and patient representatives, which should be elaborated when assessed.

**Understanding of diagnostic process** is a PRE domain for post-encounter assessment of patient’s understanding of the process [10] and, as noted by our experts, aligns with meaningful shared decision making in diagnosis and diagnostic co-production. Thus, the immediately post-encounter assessment of this domain was suggested to support and inform those efforts embracing a distributed paradigm for diagnostic work, invoking a shared mental model and collective intelligence [37].

**Experience of diagnostic uncertainty** is a PRE domain of emotions and feelings that patients might assess immediately post-encounter [31]. As note by our experts, experience of diagnostic uncertainty is inextricably connected with the patient’s own tolerance of uncertainty, and those can be assessed in tandem.

**Disconnected diagnostic encounters** across settings and providers is a PRE domain that allows patients and care partners to reflect on the coordination of care (or lack thereof) throughout the diagnostic process. One qualitative study formulated this diagnostic journey as a “pathway until you were diagnosed with cancer” and inquired if a dedicated healthcare professional had oversight and responsibility for that pathway [38]. As noted by our experts and literature, this domain might include and assess the connectedness and coordination of diagnostic process with treatment [39, 40].

**Future attitudes and behaviors toward health systems and providers** is a PRO domain that captures how the diagnostic journey affected patient confidence, faith, and trust in healthcare for future interactions. In contrast with disengagement with health systems, this domain focuses on the future, and can be illustrated with a patient’s quote: “I just don’t like doctors anymore” [27].

**Potential harms and their potential severity** is a PRO domain that assesses how a patient perceives harm that could have occurred but was prevented, including the magnitude of its potential impact on them. It was suggested that this domain might be captured similarly to the three PRO domains of harm while the possibility of harm can be stratified, for example, from “Possibly Yes” to “Definitely No” [41].

### Domains that are conceptualized but not assessed

**Awareness of pending diagnosis** was conceptualized as a potential PRE domain. While the domains of understanding of the diagnostic process and perception of a care plan to follow might include patient awareness of specific tests that are needed, experts suggested this domain might stand on its own. As patients and care partners traverse from one setting, institution, and provider to another, health systems often must rely on patients to coordinate different diagnostic procedures and ensure continuity. In those cases, patients and care partners should be aware of such roles and specifically that the results of a diagnostic procedure are pending, and that awareness can be assessed and confirmed immediately post-encounter.

**Self-advocacy affirmation** was conceptualized by experts as a potential PRO domain to capture cross-sectionally patient confidence that they can advocate for their own health throughout their diagnostic journey. It was conceptualized that patients might become less involved in their health (diminished self-advocacy) or more involved, or that advocating might become so domineering that it harms the patient’s well-being. This domain construct focuses on self-advocacy itself, making it separate from future attitudes and behaviors toward health systems and providers, and any disengagement from the health systems.

## Discussion

We identified 41 diagnostically relevant PRO and PRE domains, though this list is not intended to be exhaustive. Nor is it intended to be conceptually crisp at this stage of bringing together three streams of inquiry: an emerging construct of diagnostic excellence, diagnosis as a process and outcome that contributes to health-related quality of life, and international interest in patient-reported measurement as a tool for achieving diagnostic excellence. Our exploration at the domain level provides a foundational approach to advancing patient-reported measures for the emerging and important broad construct of diagnostic excellence.

A large number of domains presents a challenge for measurement and prioritization. Only 19 of the domains have been captured using existing instruments. More than half of the domains would need to progress from conceptualization and description in qualitative studies to more rigorous measurement. Assessing the 27 domains where patients reflect on a specific diagnostic encounter immediately and directly after that encounter would allow more immediate opportunities to act upon that reporting. The remaining 14 domains require patients to reflect on their experiences or outcomes over the entire diagnostic journey, cross-sectionally. Thus, these two groups of domains introduce different timing and setting junctures for assessment. Importantly, our findings demonstrate both substantive differences and overlaps among the domains that future measure developers would face.

In response to the challenges of such a large number of domains and relative recency of diagnostic excellence as a broadly defined construct [3], some of our experts suggested that some of the identified domains should be treated as definitely constituting patient-reported diagnostic excellence, while others as describing contributors to diagnostic excellence. However, most experts embraced the multi-domain nature of patient-reported diagnostic excellence, emphasizing, for instance, that communication-focused domains are not only contributors or risk factors to diagnostic excellence but equally constitute diagnostic excellence [44, 45]. Thus, the identified domains were seen as evolving building blocks for the emerging construct of diagnostic excellence that is being formed inductively versus deductively, especially from those emphasizing the patient perspective.

Some of our experts questioned the benefits of considering diagnostic concerns separately from other problems patients face, such as treatment concerns. Given the iterative nature of diagnosis even with empiric treatment phases, teasing out problems specific to the diagnostic process itself can be challenging. On the other hand, diagnosis has its uniqueness and emphasis; it is the foundation of the relationship a person will have with the health system [1, 12]. Additionally, the diagnostic process is often overlooked [42], and it is important to distinguish between diagnostic excellence and treatment excellence, especially given a framing of diagnostic excellence as condition-agnostic and prospectively focused.

This study also suggests care partner reporting options for identified domains following similar developments in broader patient reporting [43, 44]. Family members and other partners can help fill out patient reports in certain circumstances, such as when the patient is experiencing distress during encounters or when patients are dealing with homelessness or some types of trauma or institutional betrayal [45].

### Future directions and implications

This study underscores the need to develop definitions for identified domains and create and validate item banks for domains yet unmeasured. Another possible future direction is to review domain classification as either PRO or PRE, given potential impact on measurement approaches (e.g., distress measured as an outcome of the diagnostic process versus as an experience at varying time points during the diagnostic process). Assessment of the domains and amassing data will allow establishing how domains relate to each other under the overarching construct of diagnostic excellence. This iteration between construct definition development and attempts to capture a construct is common. Recognizing the iterative nature of doing so at the domain level for an emerging broad construct will also lead to better understanding of how to prioritize domains for further assessment, and how to utilize them to explicate the overall meaning of diagnostic excellence. For example, efforts might be made to map the identified domains into the six aims used for the initial definition of diagnostic excellence [3]. All these directions will also highlight the important role of patient-reporting for improving all aspects of patients’ diagnostic experiences and outcomes and in assessing effectiveness and efficiency of diagnostic care on patients’ well-being.

### Limitations

Our methods did not allow us to systematically identify all relevant domains or comprehensively supplement domains with many examples. Instead, we aimed to survey and assemble domains and associated measurement opportunities and challenges to facilitate future research. We might have missed existing instruments for domains, thus misclassifying them, for example, as captured only via qualitative studies. Our scope precludes providing answers to measure users on instruments to choose. However, given the relative novelty of diagnostic excellence, this exploratory analysis is expected to inform and guide measure developers on where to start. Additionally, this work did not include reports from patients on, for example, perceptions of contributors to diagnostic errors that are neither patient outcomes nor patient experiences. These patient-reported breakdowns [36] might inform diagnostic excellence but, arguably, not fully constitute the construct of patient-reported diagnostic excellence. As this construct and field matures, updates to this exploratory analysis will be needed to further systematize patient-centered assessment possibilities and realities.

## Conclusions

As the construct of diagnostic excellence matures, at least 41 patient-reported domains can be identified and contribute to inductive development of this construct. As the existing domains are both substantively different and overlapping, further research is needed to continue to iterate on domain definitions and development of instruments to assess those domains. Capturing these domains via patients’ and care partners’ reporting allows maintaining diagnostic-sharp focus on care aspects that are condition-agnostic, prospective, and impact patients’ well-being.

## Electronic supplementary material

Below is the link to the electronic supplementary material.

Supplementary Material 1

Supplementary Material 2

## Data Availability

Materials will be made available upon request to corresponding author.
