# Supplementary material for: Patient-reported outcome and experience domains for diagnostic excellence: a scoping review to inform future measure development
Source: Qual Life Res. 2024 Jun 8;33(11):2883–97. doi: 10.1007/s11136-024-03709-w (PMC11541312; doi:10.1007/s11136-024-03709-w)
Supplement: Supplementary file 2 — Supplementary Material 2 [file 11136_2024_3709_MOESM2_ESM.docx]

**Supplemental information**

**Scoping review context**

The scoping review was developed as an input to a foundation-setting project with the following aims to explore what is known about patient reporting relevant to the emerging field of “diagnostic excellence” (a relatively new construct centered on the diagnostic process and its health-related outcomes):

- Assess the state of the science on existing patient reported measures pertaining to diagnosis and define areas of gaps in measurement.
- **Identify domains relevant to the diagnostic process and associated health-related outcomes to support development of de novo patient reported measures to assess diagnostic excellence from the patient perspective.**
- Create a roadmap to guide development of patient reported measures to improve diagnosis and identify areas of promise as well as areas to avoid.

**Scoping review key questions**

- **What is the scope of patient-reported outcome (PRO) and patient-reported experience (PRE) domains that are diagnostically relevant, regardless of the future diagnosed condition?**
- **What is the state of measurement of these patient-reported domains?**

**Search terms used and search yields**

EMBASE^TM^ (as of June 12, 2020)

| **Tag** | **Search string** | **Overall** | - **2016-2020** | - **2016** | - **2017** | - **2018** | - **2019** | **2020** |
| --- | --- | --- | --- | --- | --- | --- | --- | --- |
| Emtree | 'patient-reported outcome'/**exp** | 22,633 | 21,840 | 2,107 | 4,648 | 5,499 | 6,631 | 2,955 |
| Emtree+ *(extended from Emtree)* | ('patient-reported outcome'/**exp** OR 'patient-reported' OR 'patient reported' OR 'self-report' OR 'self report' OR 'self-assess' OR 'self assess') | 206,153 | 82,518 | 16,031 | 17,749 | 19,457 | 20,709 | 8,572 |
| Emtree++ *(extended from Emtree)* | ('patient-reported outcome'/**exp** OR 'patient-reported' OR 'patient reported' OR 'patient_s reported' OR 'patients_ reported' OR 'patient experience' OR 'patient experiences' OR 'patients_ experience' OR 'patients_ experiences' OR 'patient_s experience' OR 'patient_s experiences' OR 'patient perception' OR 'patient perceptions' OR 'patients_ perception' OR 'patients_ perceptions' OR 'patient_s perception' OR 'patient_s perceptions’ OR 'patient reflection' OR 'patient reflections' OR 'patients_ reflection' OR 'patients_ reflections' OR 'patient_s reflection' OR 'patient_s reflections' OR 'patient perspective’ OR 'patient perspectives' OR 'patients_ perspective' OR 'patients_ perspectives' OR 'patient_s perspective' OR 'patient_s perspectives') | 166,354 | 74,592 | 13,725 | 16,282 | 17,619 | 19,274 | 7,692 |
| SR  and Emtree++ | ('systematic review'/**exp** OR 'systematic review' OR 'metaanalysis'/**exp** OR 'meta-analysis')  + Emtree++ | - 7,170 | 4,664 | 727 | 973 | 1127 | 1244 | 593 |
| Diagnosis  and Emtree++ | ('diagnosis'/**exp** OR diagnosis OR diagnostic)  + Emtree++ | 55,409 | 25,206 | 4,531 | 5,737 | 6,033 | 6,521 | 2,385 |
| Screening  and Emtree++ | ('screening'/**exp** OR 'screen')  + Emtree++ | 5,111 | 2,087 | 543 | 505 | 427 | 455 | 157 |
| Usability  and Emtree++ | (usability OR practical OR applicable OR applicability OR 'applicability'/**exp)**  + Emtree++ | 4,921 | 2,488 | 479 | 512 | 535 | 700 | 262 |
| Caregiver  and Emtree++ | ('caregiver'/**exp** OR 'care giver' OR 'caregiver' OR 'carer')  + Emtree++ | 3,686 | 2,005 | 345 | 447 | 494 | 528 | 191 |
| Quality improvement  and Emtree++ | ('total quality management'/**exp** OR 'quality improvement' OR 'quality management')  + Emtree++ | 2,918 | 1,669 | 309 | 349 | 393 | 457 | 161 |
| Framework  and Emtree++ | (framework OR 'conceptual')  + Emtree++ | 4,661 | 2,417 | 448 | 538 | 582 | 600 | 249 |
| Stakeholder  and Emtree++ | Stakeholder  + Emtree++ | 613 | 465 | 65 | 94 | 121 | 127 | 58 |

**Procedure to review articles from the table above**

Articles at the title/abstract stage were accumulated as the review proceeded from top to bottom rows starting with the tag “SR and Emtree++”, as well as moving from the right side of the table to the left (reverse chronological order). The approach to the title/abstract stage was directed by saturation of the unit of analysis (domains rather than articles). The reviewer stopped searching a row when new domains were not found, and moved to the next row. If the intent of the scoping review had been to achieve an exhaustive list of domains and a count of all articles capturing the identified domains, then a systematic search would have involved review of all cells and more review resources would have been required.

**Flow chart of selection of sources**

Records identified through searching EMBASE^TM^

(n=4,463)

Included:

31 sources describing

41 diagnostically relevant

PRO and PRE domains

Records screened

(n=4,495)

Records Excluded

(n=4,389)

- no patient outcomes, experiences, reflections, perceptions, or perspectives on any aspects of patient journeys that include or could relate to the diagnostic process and its outcomes

- described domain is specific to a symptom or condition

Retrieved for full text review

(n=106)

Additional record identified through other sources:

internal expert consultations (n=13)

expert convening recommendations (n=19)

Records Excluded

(n=75)

- no additional descriptions of diagnostically relevant PRO or PRE domains

- no examples of measurement tools of identified domains

**National and international organizations concerned with patient-reported measures that were searched for grey literature:**

- Australian Commission on Safety and Quality in Healthcare;
- US Department of Health and Human Services’ Patient-Reported Outcomes Measurement Information System (PROMIS);
- Organization for Economic Cooperation and Development (OECD) Patient-Reported Indicators Survey Initiative;
- Standard Sets of the International Consortium for Health Outcomes Measurement (ICHOM);
- Patient-Centered Outcomes Research Institute (PCORI);
- National Quality Forum (NQF).

**Examples of using diagnostic journey snapshots**

| **Use Case 1*: Cognitive Error – Missed Subtle Clinical Findings**  **Snapshot One (1.1)**  **OVERVIEW OF CASE**  A 55-year old man with a history of hypertension presents to a busy ED with vertigo (i.e., a sensation of movement) and vomiting for three hours since awakening. Of note, the ED has recently been focusing on improving throughput and reducing waiting times. On examination, the patient has left-beating nystagmus (i.e., uncontrolled, rapid eye movements) that changes to slight right beating when looking right, which goes undetected. These are subtle eye findings that are an indicator of stroke that go undetected by the clinician. The patient **has difficulty walking** but is able to ambulate. The neurological examination is otherwise normal. However, a Head Impulse Nystagmus Test of Skew (HINTS) examination—which would have helped detect this subtle finding—was not completed because the clinician had not been taught how to conduct this exam. A non-contrast head CT is performed that demonstrates no acute stroke. The patient **improves somewhat** with oral meclizine, which is used to help reduce vertigo symptoms. **The family voices concern that the patient is having a lot of trouble with balance**, which is **dismissed** by the team. The ED diagnosis is peripheral vertigo (i.e., labyrinthitis) which is a diagnostic error, and the patient is discharged on meclizine treatment and instructed to follow up with his primary care physician (PCP) in two to three days. The patient returns to the same hospital the next day and sees a different clinician in the ED. The patient **receives the correct diagnosis** of hemiplegia from a progressive brainstem stroke. The original diagnosing physician is never informed of the new, accurate diagnosis. | [Post-encounter domains]: remaining health concerns; satisfaction with diagnostic encounter; emotional distress during diagnostic journey; feeling of being reassured; care partner involvement in diagnostic process; perception of the symptom change trajectory.  [Subsequent domain] Inaccurate diagnosis |
| --- | --- |
| **Use Case 2*: Systems Error – Communication Failure**  **Snapshot Two (2.2)**  **OVERVIEW OF CASE**  A 70-year old Spanish-speaking female with atrial fibrillation (i.e., irregular heartbeat) on apixaban is admitted to a surgical service with appendicitis diagnosed on CT scan. Given the early stage nature of the appendicitis and the complicating challenge that she is on anticoagulants, she is treated with antibiotics as opposed to operatively. She clinically recovers after three days. However, on the CT report, a follow-up CT is suggested at three months to ensure resolution of the radiographic finding. The surgeon communicates this to the patient in non-fluent Spanish without a formal interpreter, and the surgeon assumes that the patient’s PCP will order the follow-up test. The patient **nods but does not understand**, and she **does not speak up** because she **does not wish to offend the surgeon**. The discharge follow-up instructions are printed in English, rather than Spanish, and the **patient cannot understand what is written**. The PCP sees the report and assumes that the surgeon will order the test and follow-up with the patient. Two years later, the patient is diagnosed with large appendiceal carcinoma that has **metastasized to the liver**. | [Post-encounter domains] remaining health concerns; satisfaction with diagnostic encounter; emotional distress during diagnostic journey; perception of the symptom change trajectory.  Mitigating language barriers  Interpersonal adaptation  Providers’ checking of patient understanding  Awareness of pending diagnosis  [Subsequent domain] Harm; severity of harm. |

*Snapshots are from the 2020 National Quality Forum. Improving Diagnostic Quality and Safety/Reducing Diagnostic Error: Measurement Considerations. Final Report.

**Expert convening participants**

|  | **Participant** | **Primary Affiliation** | **Primary Research Interests** |
| --- | --- | --- | --- |
|  | **Aiyegbusi, Olalekan Lee, MBChB, PhD** | University of Birmingham, UK | Management of chronic conditions, Patient-reported outcomes, Patient and public involvement and engagement |
|  | **Anhang-Price, Rebecca, PhD** | RAND Corporation, USA | Health Care Quality Measurement, Patient and family care experiences, Program Evaluation |
|  | **Beaton, Dorcas, PhD** | Institute for Work & Health, Toronto, Canada | Measurement, Translation of measurement into clinical practice, Musculoskeletal disorders |
|  | **Cleary, Paul D., PhD** | Yale School of Public Health, USA | Methods for using patient reports to improve the quality of medical care, Relationships between clinician and organizational characteristics and the quality of medical care |
|  | **Dahm, Mary, PhD, MA** | Australian National University, Australia | Communicating for Diagnostic Excellence, Language impact on patient safety and quality of care, Improving critical diagnostic conversations, Managing and communicating uncertainty |
|  | **Dolka, Io, MS** | Grey Zone, LLC, USA | Patient Advocacy, Patient-Clinician Communication for Diagnostic Excellence, Patient Engagement, Chronic and Rare Medical Conditions |
|  | **Etz, Rebecca S., PhD** | Virginia Commonwealth University, USA | Primary care measures, theories of primary care, practice transformation, and stakeholder engagement |
|  | **Geraedts, Max, MD** | Philipps University of Marburg, Germany | Healthcare quality and patient safety, Health Policy, Health Services Research |
|  | **Golden, Sara, PhD** | VA Portland Health Care System, USA | Patient-clinician communication, Communication around the risk of cancers, shared decision-making |
|  | **Greenhalgh, Joanne, PhD** | University of Leeds, UK | Evaluation of social programs, Healthcare Policy and Practice, Realist methods, Patient Reported Outcome Measures, Clinical decision making |
|  | **Hannawa, Annegret F., PhD** | Università della Svizzera italiana, Lugano, Switzerland | Safe communication, error prevention |
|  | **Hess, Rachel, MD, MS** | University of Utah Health System, USA | Patient-centered outcomes, Health-related quality of life, Interventions to improve the delivery of health care |
|  | **Holve, Erin, PhD** | Department of Health Care Finance, Washington DC Government, USA | Health Policy and Health Services Research |
|  | **Langhinrichsen-Rohling, Jennifer, PhD** | University of North Carolina, Charlotte, USA | Community-based research, Underserved and disadvantaged populations, Integration of mental and behavioral health care into primary care and school settings; Institutional betrayal and Institutional integrity |
|  | **Lundberg, Brita, MD** | Lundberg Health Advocates, USA | Patient advocacy, Infectious diseases, Diagnostic dilemmas |
|  | **Reeve, Bryce, PhD** | Duke University School of Medicine, USA | Patient-reported outcomes, Health-related Quality of Life, Cancer Outcomes, Psychometrics, and Questionnaire design |
|  | **Rendle, Katharine A., PhD, MSW, MPH** | Hospital of the University of Pennsylvania, USA | Cancer prevention and care, Healthcare delivery research, Mixed-methods research, Clinical epidemiology, Implementation science |
|  | **Repp, Allen B., MD, MSc** | University of Vermont’s Larner College of Medicine, USA | Healthcare quality and patient safety, quality measures, Hospital Medicine, General Internal Medicine |
|  | **Samuel-Ryals, Cleo A., PhD** | University of North Carolina at Chapel Hill, USA | Disentangling multilevel sources of disparities in palliative and supportive cancer care, Addressing inequities through system-level approaches, Leveraging health informatics tools |
|  | **Sandhu, Alexander, MD, MS** | Stanford Medicine, USA | Health Economics, Implementation of High-value Care Strategies, and Comparative Effectiveness |
|  | **Schwartz, Bradley L., JD** | Greater National Advocates, USA | Promoting the lifesaving benefits of independent patient advocacy in the clinical setting |
|  | **Thompson, Cristina J., MS** | University of Wollongong, Australia | Public Health, Health Services Research, Evaluation, Knowledge translation, Implementation science |
|  | **Valderas, Jose M., MD, PhD, MPH** | University of Exeter Medical School, UK | Patient reports of experiences and outcomes for decision making in clinical practice and health policy, Health care quality and safety, Primary care |
|  | **Williams, Kate, PhD** | University of Wollongong, Australia | Health and social program evaluation; health outcomes measurement; health services research |

**Sources reviewed in full-text that were excluded at that review stage not used in descriptions of identified domains or their assessments:** [1-74]

1. Abuatiq, A., Brown, R., Wolles, B., & Randall, R. (2020). Perceptions of Stress: Patient and Caregiver Experiences With Stressors During Hospitalization. Clinical journal of oncology nursing, 24(1), 51-57, doi:10.1188/20.CJON.51-57.
2. Adams, D. R., Flores, A., Coltri, A., Meltzer, D. O., & Arora, V. M. (2016). A Missed Opportunity to Improve Patient Satisfaction? Patient Perceptions of Inpatient Communication With Their Primary Care Physician. American Journal of Medical Quality : The Official Journal of the American College of Medical Quality, 31(6), 568-576, doi:10.1177/1062860615593339.
3. Addario, B., Geissler, J., Horn, M. K., Krebs, L. U., Maskens, D., Oliver, K., et al. (2020). Including the patient voice in the development and implementation of patient-reported outcomes in cancer clinical trials. Health Expectations: An International Journal of Public Participation in Health Care and Health Policy, 23(1), 41-51, doi:10.1111/hex.12997.
4. Aiyegbusi, O. L. (2020). Key methodological considerations for usability testing of electronic patient-reported outcome (ePRO) systems. Quality of life research : an international journal of quality of life aspects of treatment, care and rehabilitation, 29(2), 325-333, doi:10.1007/s11136-019-02329-z.
5. Anderson, L. M., Papadakis, J. L., Vesco, A. T., Shapiro, J. B., Feldman, M. A., Evans, M. A., et al. (2020). Patient-Reported and Parent Proxy-Reported Outcomes in Pediatric Medical Specialty Clinical Settings: A Systematic Review of Implementation. Journal of pediatric psychology, 45(3), 247-265, doi:10.1093/jpepsy/jsz082.
6. Anhang Price, R., Elliott, M. N., Zaslavsky, A. M., Hays, R. D., Lehrman, W. G., Rybowski, L., et al. (2014). Examining the role of patient experience surveys in measuring health care quality. Medical care research and review: MCRR, 71(5), 522-554, doi:10.1177/1077558714541480.
7. Bartley, N., Napier, C., Best, M., & Butow, P. (2020). Patient experience of uncertainty in cancer genomics: a systematic review. Genetics in medicine : official journal of the American College of Medical Genetics, 22(9), 1450-1460, doi:10.1038/s41436-020-0829-y.
8. Basch, E., Deal, A. M., Kris, M. G., Scher, H. I., Hudis, C. A., Sabbatini, P., et al. (2016). Symptom Monitoring With Patient-Reported Outcomes During Routine Cancer Treatment: A Randomized Controlled Trial. Journal of clinical oncology : official journal of the American Society of Clinical Oncology, 34(6), 557-565, doi:10.1200/JCO.2015.63.0830.
9. Basch, E., Spertus, J., Dudley, R. A., Wu, A., Chuahan, C., Cohen, P., et al. (2015). Methods for Developing Patient-Reported Outcome-Based Performance Measures (PRO-PMs). Value in health : the journal of the International Society for Pharmacoeconomics and Outcomes Research, 18(4), 493-504, doi:10.1016/j.jval.2015.02.018.
10. Basch, E., Wilfong, L., & Schrag, D. (2020). Adding Patient-Reported Outcomes to Medicare's Oncology Value-Based Payment Model. Jama, 323(3), 213-214, doi:10.1001/jama.2019.19970.
11. Beardon, S., Patel, K., Davies, B., & Ward, H. (2018). Informal carers’ perspectives on the delivery of acute hospital care for patients with dementia: a systematic review. BMC Geriatrics, 18(1), 23, doi:10.1186/s12877-018-0710-x.
12. Berning, M. J., Oliveira J E Silva, L., Suarez, N. E., Walker, L. E., Erwin, P., Carpenter, C. R., et al. (2020). Interventions to improve older adults' Emergency Department patient experience: A systematic review. The American Journal of Emergency Medicine, 38(6), 1257-1269, doi:10.1016/j.ajem.2020.03.012.
13. Bhatnagar, V., Hudgens, S., Piault-Louis, E., Jones, L., Beaver, J. A., Lyerly, H. K., et al. (2020). Patient-Reported Outcomes in Oncology Clinical Trials: Stakeholder Perspectives from the Accelerating Anticancer Agent Development and Validation Workshop 2019. The oncologist, 25(10), 819-821, doi:10.1634/theoncologist.2020-0062.
14. Biber, J., Ose, D., Reese, J., Gardiner, A., Facelli, J., Spuhl, J., et al. (2018). Patient reported outcomes - experiences with implementation in a University Health Care setting. Journal of patient-reported outcomes, 2, 34-0. eCollection 2017, doi:10.1186/s41687-018-0059-0.
15. Black, K. Z., Lightfoot, A. F., Schaal, J. C., Mouw, M. S., Yongue, C., Samuel, C. A., et al. (2021). 'It's like you don't have a roadmap really': using an antiracism framework to analyze patients' encounters in the cancer system. Ethnicity & Health, 26(5), 676-696, doi:10.1080/13557858.2018.1557114.
16. Bontempo, A. C., & Mikesell, L. (2020). Patient perceptions of misdiagnosis of endometriosis: results from an online national survey, 7(2), 97-106, doi:10.1515/dx-2019-0020.
17. Boyce, M. B., Browne, J. P., & Greenhalgh, J. (2014). The experiences of professionals with using information from patient-reported outcome measures to improve the quality of healthcare: a systematic review of qualitative research. BMJ quality & safety, 23(6), 508-518, doi:10.1136/bmjqs-2013-002524.
18. Boylan, A., Turk, A., van Velthoven, M. H., & Powell, J. (2020). Online patient feedback as a measure of quality in primary care: a multimethod study using correlation and qualitative analysis. BMJ open, 10(2), e031820-031820, doi:10.1136/bmjopen-2019-031820.
19. Briggs, M. S., Rethman, K. K., Crookes, J., Cheek, F., Pottkotter, K., McGrath, S., et al. (2020). Implementing Patient-Reported Outcome Measures in Outpatient Rehabilitation Settings: A Systematic Review of Facilitators and Barriers Using the Consolidated Framework for Implementation Research. Archives of Physical Medicine and Rehabilitation, 101(10), 1796-1812, doi:10.1016/j.apmr.2020.04.007.
20. Centor, R. M., Geha, R., & Manesh, R. (2019). The Pursuit of Diagnostic Excellence. JAMA Network Open, 2(12), e1918040, doi:10.1001/jamanetworkopen.2019.18040.
21. Cheraghi-Sohi, S., Holland, F., Singh, H., Danczak, A., Esmail, A., Morris, R. L., et al. (2021). Incidence, origins and avoidable harm of missed opportunities in diagnosis: longitudinal patient record review in 21 English general practices. BMJ quality & safety, 30(12), 977-985, doi:10.1136/bmjqs-2020-012594.
22. Clapham, S., Daveson, B. A., Allingham, S. F., Morris, D., Blackburn, P., Johnson, C. E., et al. (2021). Patient-reported outcome measurement of symptom distress is feasible in most clinical scenarios in palliative care: an observational study involving routinely collected data. International journal for quality in health care : journal of the International Society for Quality in Health Care, 33(2), mzab075. doi: 10.1093/intqhc/mzab075, doi:10.1093/intqhc/mzab075.
23. Dako, F., Wray, R., Awan, O., & Subramaniam, R. M. (2017). Adapting a Standardized, Industry-Proven Tool to Measure Patients' Perceptions of Quality at the Point of Care in a PET/CT Center. Journal of nuclear medicine technology, 45(4), 285-289, doi:10.2967/jnmt.117.196170.
24. Danforth, K. N., Smith, A. E., Loo, R. K., Jacobsen, S. J., Mittman, B. S., & Kanter, M. H. (2014). Electronic Clinical Surveillance to Improve Outpatient Care: Diverse Applications within an Integrated Delivery System. EGEMS (Washington, DC), 2(1), 1056-9214.1056. eCollection 2014, doi:10.13063/2327-9214.1056.
25. De Rosis, S., Cerasuolo, D., & Nuti, S. (2020). Using patient-reported measures to drive change in healthcare: the experience of the digital, continuous and systematic PREMs observatory in Italy. BMC Health Services Research, 20(1), 315, doi:10.1186/s12913-020-05099-4.
26. Edmondson, A. J., Birtwistle, J. C., Catto, J. W. F., & Twiddy, M. (2017). The patients' experience of a bladder cancer diagnosis: a systematic review of the qualitative evidence. Journal of cancer survivorship : research and practice, 11(4), 453-461, doi:10.1007/s11764-017-0603-6.
27. Elliott, M. N., Beckett, M. K., Lehrman, W. G., Cleary, P., Cohea, C. W., Giordano, L. A., et al. (2016). Understanding The Role Played By Medicare's Patient Experience Points System In Hospital Reimbursement. Health affairs (Project Hope), 35(9), 1673-1680, doi:10.1377/hlthaff.2015.0691.
28. Epstein, R. M., & Street, R. L., Jr. (2007). Patient-Centered Communication in Cancer Care: Promoting Healing and Reducing Suffering. Bethesda, MD: National Cancer Institute.
29. Etz, R. S., Zyzanski, S. J., Gonzalez, M. M., Reves, S. R., O'Neal, J. P., & Stange, K. C. (2019). A New Comprehensive Measure of High-Value Aspects of Primary Care. Annals of Family Medicine, 17(3), 221-230, doi:10.1370/afm.2393.
30. Farias, A. J., Ochoa, C. Y., Toledo, G., Bang, S., Hamilton, A. S., & Du, X. L. (2020). Racial/ethnic differences in patient experiences with health care in association with earlier stage at breast cancer diagnosis: findings from the SEER-CAHPS data. Cancer causes & control : CCC, 31(1), 13-23, doi:10.1007/s10552-019-01254-3.
31. Farley, H., Enguidanos, E. R., Coletti, C. M., Honigman, L., Mazzeo, A., Pinson, T. B., et al. (2014). Patient satisfaction surveys and quality of care: an information paper. Annals of Emergency Medicine, 64(4), 351-357, doi:10.1016/j.annemergmed.2014.02.021.
32. Gelkopf, M., Mazor, Y., & Roe, D. (2021). A systematic review of patient-reported outcome measurement (PROM) and provider assessment in mental health: goals, implementation, setting, measurement characteristics and barriers. International journal for quality in health care : journal of the International Society for Quality in Health Care, 34(Suppl 1), ii13–ii27, doi:10.1093/intqhc/mzz133.
33. Gustafsson, N., Leino-Kilpi, H., Prga, I., Suhonen, R., Stolt, M., & RANCARE consortium COST Action – CA15208. (2020). Missed Care from the Patient's Perspective - A Scoping Review. Patient preference and adherence, 14, 383-400, doi:10.2147/PPA.S238024.
34. Guyatt, G. H., Kirshner, B., & Jaeschke, R. (1992). Measuring health status: what are the necessary measurement properties?. Journal of clinical epidemiology, 45(12), 1341-1345, doi:10.1016/0895-4356(92)90194-r.
35. Haldar, S., Mishra, S. R., Pollack, A. H., & Pratt, W. (2020). Informatics opportunities to involve patients in hospital safety: a conceptual model. Journal of the American Medical Informatics Association : JAMIA, 27(2), 202-211, doi:10.1093/jamia/ocz167.
36. Hannawa, A. F. (2020). The mind of an academic, the voice of a patient: My field experience with safe communication. Journal of Patient Safety and Risk Management, 25(4), 144-146, doi:10.1177/2516043520913409.
37. Hargraves, J. L., Cosenza, C., Elliott, M. N., & Cleary, P. D. (2019). The effect of different sampling and recall periods in the CAHPS Clinician & Group (CG-CAHPS) survey. Health services research, 54(5), 1036-1044, doi:10.1111/1475-6773.13173.
38. Institute of Medicine (US) Committee on Future Directions for the National Healthcare Quality and Disparities Reports. (2010). Future Directions for the National Healthcare Quality and Disparities Reports. Washington (DC): National Academies Press (US).
39. Institute of Medicine (US) Committee on Understanding and Eliminating Racial and Ethnic Disparities in Health Care; Smedley BD, Stith AY, Nelson AR, editors. Unequal Treatment: Confronting Racial and Ethnic Disparities in Health Care. Washington (DC): National Academies Press (US); 2003. Available from: https://www.ncbi.nlm.nih.gov/books/NBK220358/ doi: 10.17226/12875
40. Kalluri, M., Luppi, F., & Ferrara, G. (2020). What Patients With Idiopathic Pulmonary Fibrosis and Caregivers Want: Filling the Gaps With Patient Reported Outcomes and Experience Measures. The American Journal of Medicine, 133(3), 281-289, doi:10.1016/j.amjmed.2019.08.032.
41. Lee, J. M., Lowry, K. P., Cott Chubiz, J. E., Swan, J. S., Motazedi, T., Halpern, E. F., et al. (2020). Breast cancer risk, worry, and anxiety: Effect on patient perceptions of false-positive screening results. Breast (Edinburgh, Scotland), 50, 104-112, doi:10.1016/j.breast.2020.02.004.
42. Lee, J., Lee, E., Chae, D., & Kim, C. (2020). Patient-reported outcome measures for diabetes self-care: A systematic review of measurement properties. International journal of nursing studies, 105, 103498, doi:10.1016/j.ijnurstu.2019.103498.
43. MacDonald, S., Sampson, C., Turley, R., Biddle, L., Ring, N., Begley, R., et al. (2020). Patients' Experiences of Emergency Hospital Care Following Self-Harm: Systematic Review and Thematic Synthesis of Qualitative Research. Qualitative health research, 30(3), 471-485, doi:10.1177/1049732319886566.
44. Maini, R., Kirkpatrick, M., McCafferty, A., Dunkley, C., Ogston, S., & Williams, F. (2018). Evaluation of a questionnaire to measure parent/carer and child/young person experience of NHS epilepsy services. Seizure, 63, 71-78, doi:10.1016/j.seizure.2018.11.002.
45. Mann, C. M., Schanberg, L. E., Wang, M., von Scheven, E., Lucas, N., Hernandez, A., et al. (2020). Identifying clinically meaningful severity categories for PROMIS pediatric measures of anxiety, mobility, fatigue, and depressive symptoms in juvenile idiopathic arthritis and childhood-onset systemic lupus erythematosus. Quality of life research : an international journal of quality of life aspects of treatment, care and rehabilitation, 29(9), 2573-2584, doi:10.1007/s11136-020-02513-6.
46. McLachlan, S., Clements, A., & Austoker, J. (2012). Patients' experiences and reported barriers to colonoscopy in the screening context--a systematic review of the literature. Patient education and counseling, 86(2), 137-146, doi:10.1016/j.pec.2011.04.010.
47. Morreale, M. K., Moore, T. F., Kim, S., Uphold, H. S., Mabunda, L. M., & Harper, F. W. K. (2020). Preferences for notification of imaging results in patients with metastatic cancer. Patient education and counseling, 103(2), 392-397, doi:10.1016/j.pec.2019.08.032.
48. Neilson, L. J., Patterson, J., von Wagner, C., Hewitson, P., McGregor, L. M., Sharp, L., et al. (2020). Patient experience of gastrointestinal endoscopy: informing the development of the Newcastle ENDOPREM™. Frontline gastroenterology, 11(3), 209-217, doi:10.1136/flgastro-2019-101321.
49. Nic Giolla Easpaig, B., Tran, Y., Bierbaum, M., Arnolda, G., Delaney, G. P., Liauw, W., et al. (2020). What are the attitudes of health professionals regarding patient reported outcome measures (PROMs) in oncology practice? A mixed-method synthesis of the qualitative evidence. BMC health services research, 20(1), 102, doi:10.1186/s12913-020-4939-7.
50. National Quality Forum (NQF).(2020). Patient-reported outcomes: Best practices on selection and data collection. Final Technical Report. . Washington, DC; National Quality Forum:. https://www.qualityforum.org/Publications/2020/09/Patient-Reported_Outcomes__Best_Practices_on_Selection_and_Data_Collection_-_Final_Technical_Report.aspx
51. NSW Agency for Clinical Innovation. (2021). Analytic principles for patient-reported outcome measures.
52. Oldenburger, E., Oldenburger, F., Coolbrandt, A., Isebaert, S., Neyens, I., Sevenants, A., et al. (2020). The use of patient reported outcome measures (PROMs) in palliative radiotherapy: A topical review. Radiotherapy and oncology : journal of the European Society for Therapeutic Radiology and Oncology, 149, 94-103, doi:10.1016/j.radonc.2020.04.045.
53. Øvretveit, J., Zubkoff, L., Nelson, E. C., Frampton, S., Knudsen, J. L., & Zimlichman, E. (2017). Using patient-reported outcome measurement to improve patient care. International Journal for Quality in Health Care: Journal of the International Society for Quality in Health Care, 29(6), 874-879, doi:10.1093/intqhc/mzx108.
54. Ozavci, G., Bucknall, T., Woodward-Kron, R., Hughes, C., Jorm, C., Joseph, K., et al. (2021). A systematic review of older patients’ experiences and perceptions of communication about managing medication across transitions of care. Research in Social and Administrative Pharmacy, 17(2), 273-291, doi:10.1016/j.sapharm.2020.03.023.
55. Parast, L., Haas, A., Tolpadi, A., Elliott, M. N., Teno, J., Zaslavsky, A. M., et al. (2018). Effects of Caregiver and Decedent Characteristics on CAHPS Hospice Survey Scores. Journal of pain and symptom management, 56(4), 519-529.e1, doi:10.1016/j.jpainsymman.2018.07.014.
56. Pellizzoni, L., E Silva, S. d. A., & Falavigna, A. (2020). Multilanguage health record database focused on the active follow-up of patients and adaptable for patient-reported outcomes and clinical research design. International journal of medical informatics, 135, 104065, doi:10.1016/j.ijmedinf.2019.104065.
57. Quigley, D. D., Parast, L., Haas, A., Elliott, M. N., Teno, J. M., & Anhang Price, R. (2020). Differences in Caregiver Reports of the Quality of Hospice Care Across Settings. Journal of the American Geriatrics Society, 68(6), 1218-1225, doi:10.1111/jgs.16361.
58. Samuel, C. A., Smith, A. B., Elkins, W., Richmond, J., Mahbooba, Z., Basch, E., et al. (2021). Racial differences in user experiences and perceived value of electronic symptom monitoring in a cohort of black and white bladder and prostate cancer patients. Quality of life research : an international journal of quality of life aspects of treatment, care and rehabilitation, 30(11), 3213-3227, doi:10.1007/s11136-020-02442-4.
59. Schlesinger, M., Grob, R., & Shaller, D. (2015). Using Patient-Reported Information to Improve Clinical Practice. Health Services Research, 50(S2), 2116-2154, doi:10.1111/1475-6773.12420.
60. Scott, V. C., Gold, S. B., Kenworthy, T., Snapper, L., Gilchrist, E. C., Kirchner, S., et al. (2021). Assessing cross-sector stakeholder readiness to advance and sustain statewide behavioral integration beyond a State Innovation Model (SIM) initiative. Translational behavioral medicine, 11(7), 1420-1429, doi:10.1093/tbm/ibab022.
61. Scott, V. C., Kenworthy, T., Godly-Reynolds, E., Bastien, G., Scaccia, J., McMickens, C., et al. (2017). The Readiness for Integrated Care Questionnaire (RICQ): An instrument to assess readiness to integrate behavioral health and primary care. The American Journal of Orthopsychiatry, 87(5), 520-530, doi:10.1037/ort0000270.
62. Squitieri, L., Bozic, K. J., & Pusic, A. L. (2017). The Role of Patient-Reported Outcome Measures in Value-Based Payment Reform. Value in health : the journal of the International Society for Pharmacoeconomics and Outcomes Research, 20(6), 834-836, doi:10.1016/j.jval.2017.02.003.
63. Stange, K. C., Etz, R. S., Gullett, H., Sweeney, S. A., Miller, W. L., Jaén, C. R., et al. (2014). Metrics for assessing improvements in primary health care. Annual Review of Public Health, 35, 423-442, doi:10.1146/annurev-publhealth-032013-182438.
64. Stover, A. M., Haverman, L., van Oers, H. A., Greenhalgh, J., Potter, C. M., & ISOQOL PROMs/PREMs in Clinical Practice Implementation Science Work Group. (2021). Using an implementation science approach to implement and evaluate patient-reported outcome measures (PROM) initiatives in routine care settings. Quality of life research : an international journal of quality of life aspects of treatment, care and rehabilitation, 30(11), 3015-3033, doi:10.1007/s11136-020-02564-9.
65. Stover, A. M., Urick, B. Y., Deal, A. M., Teal, R., Vu, M. B., Carda-Auten, J., et al. (2020). Performance Measures Based on How Adults With Cancer Feel and Function: Stakeholder Recommendations and Feasibility Testing in Six Cancer Centers. JCO oncology practice, 16(3), e234-e250, doi:10.1200/JOP.19.00784.
66. Tan, W. S., Teo, C. H., Chan, D., Ang, K. M., Heinrich, M., Feber, A., et al. (2020). Exploring patients' experience and perception of being diagnosed with bladder cancer: a mixed-methods approach. BJU international, 125(5), 669-678, doi:10.1111/bju.15008.
67. Tomlinson, D., Yuan, C., Cheng, L., & Hinds, P. S. (2020). Patient-Reported Outcomes in Pediatric Oncology: The Voice of the Child. In P. S. Hinds, & L. Linder (Eds.), Pediatric Oncology Nursing: Defining Care Through Science (pp. 107-129). Cham: Springer International Publishing.
68. Turner, G. M., Litchfield, I., Finnikin, S., Aiyegbusi, O. L., & Calvert, M. (2020). General practitioners' views on use of patient reported outcome measures in primary care: a cross-sectional survey and qualitative study. BMC family practice, 21(1), 14-6, doi:10.1186/s12875-019-1077-6.
69. Vagal, A., Wahab, S., Lecky, S., Washburn, E., Schwartz, R., Vogel, C., et al. (2020). Optimizing Patient Experience Using Human-Centered Design. Journal of the American College of Radiology : JACR, 17(5), 668-672, doi:10.1016/j.jacr.2019.11.020.
70. Vaillancourt, S., Seaton, M. B., Schull, M. J., Cheng, A. H. Y., Beaton, D. E., Laupacis, A., et al. (2017). Patients' Perspectives on Outcomes of Care After Discharge From the Emergency Department: A Qualitative Study. Annals of Emergency Medicine, 70(5), 648-658.e2, doi:10.1016/j.annemergmed.2017.05.034.
71. Van Der Wees, P. J., Nijhuis-Van Der Sanden, M. W. G., Ayanian, J. Z., Black, N., Westert, G. P., & Schneider, E. C. (2014). Integrating the use of patient-reported outcomes for both clinical practice and performance measurement: views of experts from 3 countries. The Milbank Quarterly, 92(4), 754-775, doi:10.1111/1468-0009.12091.
72. van Nuenen, F. M., Donofrio, S. M., Tuinman, M. A., van de Wiel, H. B. M., & Hoekstra-Weebers, J. E. H. M. (2020). Effects on patient-reported outcomes of "Screening of Distress and Referral Need" implemented in Dutch oncology practice. Supportive care in cancer : official journal of the Multinational Association of Supportive Care in Cancer, 28(7), 3391-3398, doi:10.1007/s00520-019-05140-1.
73. Weiskopf, N. G., Bakken, S., Hripcsak, G., & Weng, C. (2017). A Data Quality Assessment Guideline for Electronic Health Record Data Reuse. EGEMS (Washington, DC), 5(1), 14, doi:10.5334/egems.218.
74. Williams, K. E., Sansoni, J., Morris, D., & Thompson, C. (2018). A Delphi study to develop indicators of cancer patient experience for quality improvement. Supportive care in cancer : official journal of the Multinational Association of Supportive Care in Cancer, 26(1), 129-138, doi:10.1007/s00520-017-3823-4.
75. Wray, J., & Oldham, G. (2020). Using parent-reported experience measures as quality improvement tools in paediatric cardiothoracic services: making it happen. International journal for quality in health care : journal of the International Society for Quality in Health Care, 32(2), 140-148, doi:10.1093/intqhc/mzaa001.
